# Development of a Radiomics Prediction Model for Histological Type Diagnosis in Solitary Pulmonary Nodules: The Combination of CT and FDG PET

**DOI:** 10.3389/fonc.2020.555514

**Published:** 2020-09-15

**Authors:** Mengmeng Yan, Weidong Wang

**Affiliations:** ^1^Urban Vocational College of Sichuan, Chengdu, China; ^2^Sichuan Cancer Hospital & Institute, Chengdu, China; ^3^Department of Radiation Oncology, Sichuan Cancer Hospital & Institute, Chengdu, China; ^4^Radiation Oncology Key Laboratory of Sichuan Province, Sichuan Cancer Hospital, Chengdu, China

**Keywords:** radiomics, lung cancer, histological subtypes, CT, PET

## Abstract

**Purpose:**

To develop a diagnostic model for histological subtypes in lung cancer combined CT and FDG PET.

**Methods:**

Machine learning binary and four class classification of a cohort of 445 lung cancer patients who have CT and PET simultaneously. The outcomes to be predicted were primary, metastases (Mts), adenocarcinoma (Adc), and squamous cell carcinoma (Sqc). The classification method is a combination of machine learning and feature selection that is a Partition-Membership. The performance metrics include accuracy (Acc), precision (Pre), area under curve (AUC) and kappa statistics.

**Results:**

The combination of CT and PET radiomics (CPR) binary model showed more than 98% Acc and AUC on predicting Adc, Sqc, primary, and metastases, CPR four-class classification model showed 91% Acc and 0.89 Kappa.

**Conclusion:**

The proposed CPR models can be used to obtain valid predictions of histological subtypes in lung cancer patients, assisting in diagnosis and shortening the time to diagnostic.

## Introduction

Differentiation of histological types of lung cancer is the base for its treatment. Biopsy is the most important part of diagnostic pathology. It can make clear histopathological diagnosis for the vast majority of cases, which is regarded as the final clinical diagnosis (1), but it is traumatic and costly. Radiomics is a cost-effective method to predict histological subtypes in lung cancer by using images features as the markers (2–5).

The workflow of radiomics includes image acquisition, image preprocessing, volume of interest segmentation, feature extraction, feature selection, model building and validation. Sollini et al. has comprehensively and clearly reported the methodological aspects of the radiomics workflow and possible pitfalls (2, 3). In particular, for image types, different types of medical images have different advantages. For example, CT image has higher density resolution, PET has high sensitivity and specificity, it can show the lesion when it is in the early stage of molecular level changes.

This paper tests the hypothesis that the combination of CT and PET radiomics (CPR) features has a better classification ability than CT-based radiomics (CTR) or PET-based radiomics (PETR). To invest the evidence of that, we built 24 classifiers to compare the performance of CTR, PETR, and CPR. This study is the first radiomics study combining CT and PET, it is also the first radiomics study to predict adenocarcinoma (Adc), squamous cell carcinoma (Sqc), and metastases (Mts) simultaneously (four-class classification).

## Materials and Methods

This study was approved by the institutional Ethics Committee. The tool used for statistical analysis was WEKA (Frank E. et al., presented at the 2009 Data mining and knowledge discovery handbook) (Weka v3.8.3, Hamilton, New Zealand).

### Patients

We used a public data set of radiomics features, consists of 534 patients with lung cancer (5). We selected 445 patients who have both CT and PET images, including 168 Adc, 129 Sqc, 81 Mts, and 67 other primary lung cancer types (Oth). For this data set, the patient characteristics and radiomics features are available. The inclusion criteria were: (a) age >18 years and (b) histological diagnosis of either primary or metastatic tumor obtained from CT-guided biopsy, endobronchial ultrasound-guided biopsy, videothoracoscopy or surgical removal of a lung lesion (5). The exclusion criteria were: (a) inconclusive histology from an inadequate biopsy sample, (b) diagnosis of non-malignancy, and (c) FDG uptake below or comparable to background activity within the parenchyma of the healthy lung (5).

### Image Acquisition, Segmentation, and Texture Computation

Imaging protocol and image processing approaches have been described in detail, according to the Image Biomarker Standardisation Initiative (IBSI) reporting guidelines (5). FDG PET/CT images were collected by PET/CT scanner 60 ± 5 min after injection of FDG, the fixed dose ranged from 350 to 550 MBq. PET image reconstruction methods included iterative and time of flight. The PET resolutions were 5.3 mm × 5.3 mm × 2.0 mm and 2.7 × 2.7 × 3.27, CT resolutions were 0.98 mm × 0.98 mm × 4.0 mm and 1.37 mm × 1.37 mm × 3.27 mm. PET images were corrected for attenuation using the acquired CT data, The volume of interest (VOI) of lung lesion was automatically defined on PET images, and the threshold value is 40% of the maximum standard uptake value (SUVmax) (5).

The texture features of CT and PET images under the same VOI are calculated by lifex software package[], 43 features were extracted from PET image and 41 from CT image, LIFEx package calculates texture features for VOIs of at least 64 voxels, the CT-based radiomics features were studied within 534 patients (CT datasets), the PET data set consisted of 482 patients. The average size of the lesions was 1.64 ± 0.78 cm (range 0.49–5.23 cm) (5). There are 37 features in CTR features, which are the same as PETR features. The same features include volume, geometry-based and histogram-based features, gray level co-occurrence matrix, neighborhood gray level difference matrix, gray level run length matrix, and gray level zone length matrix. CTR and PETR have different basic features.

### Analysis

#### Feature Selection and Normalization

In order to select features with good repeatability and reproducibility, and to avoid over fitting. We studied the related researches about the stability of radiomics features. According to the study results of stability and reproducibility of the radiomics features (6, 7), we selected 2 CTR features, Skewness and Kurtosis based on histogram, 2 PETR features, SUVmean and SUVmax. The 2 CTR features were assessed by compatibility ratios (>80%) based on t-test, which have a good reproducibility against slice thickness. And the 2 PETR features were assessed by meta-analysis of 21 studies, which also have a good reproducibility against slice thickness.

The selected radiomics features were normalized to a Z-score.

#### Model Building and Performance Evaluation

Firstly, the study is divided into binary classification and four-class classification experiments. Binary classification experiments include the prediction of lung adenocarcinoma from lung cancer patients (T1), the prediction of squamous cell carcinoma from lung cancer patients (T2), and the distinction between metastatic lung cancer and primary lung cancer (T3). Four-class classification experiment is used to predict the lung cancer histological type (T4), including lung adenocarcinoma, lung squamous cell carcinoma, metastatic lung cancer, and other histological types of lung cancer. Each experiment randomly divided the data set into training set and test set by 8:2, repeatedly dividing the whole data set until the distribution of the data sets is the same. Finally, set the two data sets as training set and test set. Table 1 shows the size of training set and test set for each experiment.

**TABLE 1:** Training set and test set of CT, or PET for binary and four-class classification.

	Training set	Test set
*Binary classification		
Adc vs. NAdc	131 vs. 221	37 vs. 56
Sqc vs. NSqc	103 vs. 249	26 vs. 67
Primary vs. Mts	287 vs. 65	77 vs. 16
Four-class classification		
Adc vs. Sqc vs. Mts vs. Oth	131 vs. 103 vs. 65 vs. 53	37 vs. 26 vs. 16 vs. 14

*Adc, Adenocarcinoma, Sqc, squamous cell carcinoma, Mts, metastases, NAdc, not adenocarcinoma, NSqc, not squamous cell carcinoma.

Secondly, in order to maximize the use of existing data, the data set classes should be balanced before model building. We reweighed the instances in the data so that each class has the same total weight (Classbalancer in Weka). This method can keep data balance without deleting cases.

Then the partition-Membership filter (PMF, PartitionMembershipFilter with option Random Committee in Weka) used to transform the normalized 2 PETR and 2 CTR features into sparse instances to improve the model performance (34, 35).

Finally, the transformed features were input into two machine learning classifiers, ensemble learning classifier Random Forest (RandomForest with options -K 0 -M 1.0 -V 0.001 -S 1 in Weka) and Sequential Minimal Optimization (SMO with options -C 1.0 -L 0.001 -P 1.0E-12 -N 1 -V-1 -W 1 -K in Weka) with 10-folds cross validation. The performance metrics of the classification model include accuracy (Acc), precision (Pre), area under curve (AUC) and kappa statistics.

## Results

### Data Size

Table 1 shows the data size for each model. Each classification experiment consists of 445 patients and no one deleted. NAdc (not Adc), consists of Sqc, Mts and others primary lung cancer types. NSqc (not Sqc), consists of Adc, Mts and others primary lung cancer types.

### Binary Classification Models

**FIGURE 1:**
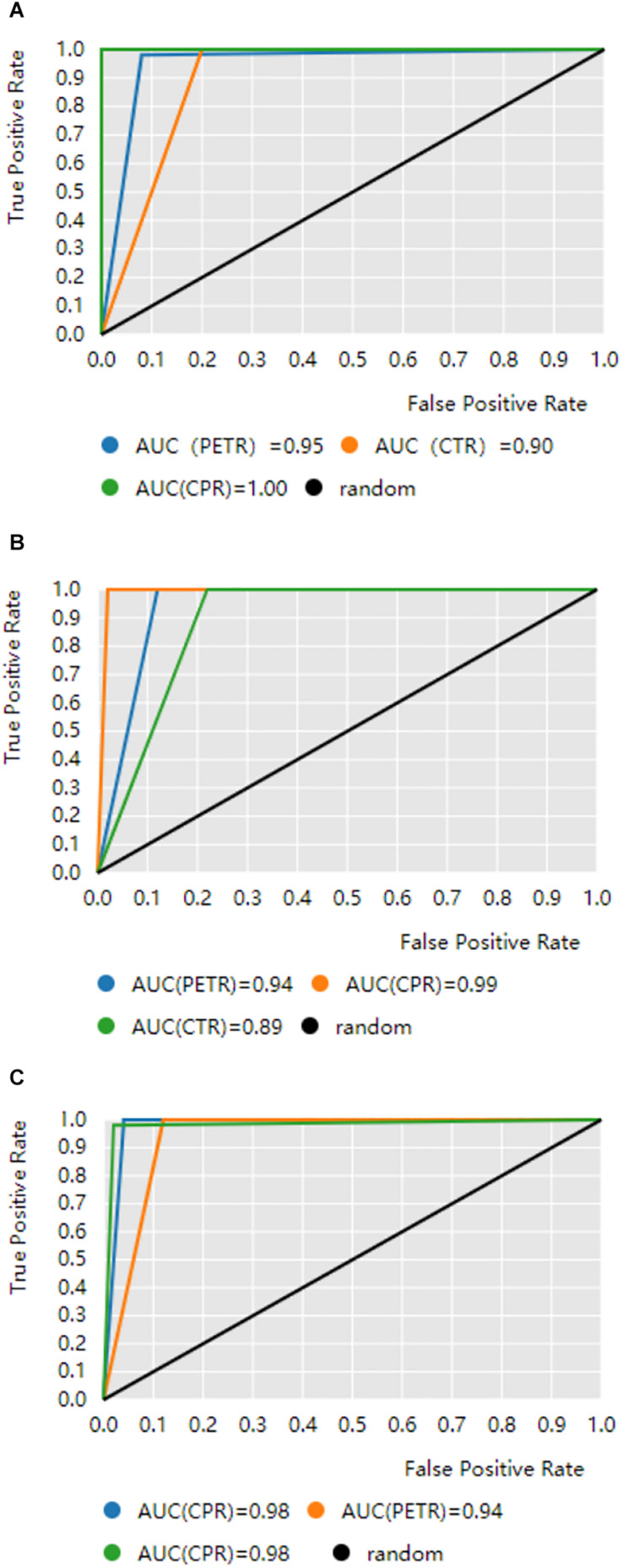
ROC curves obtained by binary classification models. The black diagonal line in the diagram is the random line which is the worst possible performance a model can achieve. CTR is CT-based radiomics, PETR is PET-based radiomics, CPR is the combination CTR and PETR. **(A)** Predicting lung adenocarcinoma from lung cancer patients. **(B)** Predicting squamous cell carcinoma from lung cancer patients. **(C)** The distinction between metastatic lung cancer and primary lung cancer.

Table 2 and Figure 1 show the results of binary Classification models on the test set. CPR has the performance on Adc/NAdc, Sqc/NSqc, and Primary/Mts. It is because the combination of CT and PET have more information than using CT or PET only. Tables 2(a) and (b) show the performance of PETR is better than CPR on Adc/NAdc and Sqc/NSqc. it can be inferred that PETR features can differentiate Adc and Sqc well (AUC >the 0.94). Table 2(c) shows CTR is better than PETR on Adc/Sqc, it can be inferred that CTR features have better performance on differentiating Pre from Mts (AUC = 0.98).

**TABLE 2:** Binary classification results on test set*.

Performance	^1^CTR (RF)	^2^PETR (RF)	^3^CPR (SMO)
**(a) Adc vs. NAdc**			
Accuracy	81.6	85.0	**100.0**
True positive rate	0.81	0.89	**1.00**
True negative rate	0.82	0.80	**1.00**
Mean of precision	0.88	0.85	**1.00**
AUC	0.90	0.95	**1.00**

**Performance**	**CTR**	**PETR**	**CPR**

**(b) Sqc vs. NSqc**			
Accuracy	76.3	83.4	**98.5**
True positive rate	0.94	0.90	**0.97**
True negative rate	0.57	0.77	**1.00**
Mean of precision	0.80	0.84	**0.99**
AUC	0.89	0.94	**0.99**
**(c) Primary vs. Mts**			
Accuracy	86.6	80.9	**98.0**
True positive rate	0.96	0.92	**0.96**
True negative rate	0.75	0.69	**1.00**
Mean of precision	0.88	0.82	**0.98**
AUC	**0.98**	0.94	**0.98**

*Adc, Adenocarcinoma, Sqc, squamous cell carcinoma, Mts, metastases, NAdc, not adenocarcinoma, NSqc, not squamous cell carcinoma. ^1^CT-based radiomics (with Random Forest classification). ^2^PET-based radiomics (with Random Forest classification). ^3^The combination of CT- and PET-based radiomics (with Sequential minimal optimization classification). The best performance metrics for each classification are highlighted in bold.

However, it is important to diagnose primary from Mts, Adc from NAdc, and Sqc from NSqc so that the patients will get treatment earlier. Table 2 shows our CPR models achieved an Acc ratio of 100% on Adc/NAdc, 97% on Sqc/NSqc, 96% on primary/Mts, which are acceptable to apply to clinical diagnosis.

### Four-Class Classification Models

Table 3 shows the model performance of predicting Adc, Sqc, and Mts simultaneously. CPR has the best performance, followed by PETR. Kappa coefficient is used to evaluate the model classification ability comprehensively, CPR performs almost perfect with the 0.89 kappa. The four-class CPR model performs well in identifying Adc, Sqc, and Mts since its true rate and precision are both high (more than 85%). Especially the Acc and primary for Mts are 100% which means all of our predictions as Mts are true Mts, and among all true Mts, our four-class model successfully predicted 100% of them. The Acc and primary of CPR are higher than that of CTR and PETR, it is reasonable since CPR combines the Identification ability CTR and PETR. Table 3 also shows PETR can show more information on expressing lung cancer Histological types.

**TABLE 3:** Four-class classification on test set.

Performance	^1^CTR (RF)	^2^PETR (RF)	^3^CPR (SMO)
Accuracy (%)	62.9	79.1	**91.2**
**True rate for:**			
Adc	0.73	**0.89**	**0.89**
Sqc	0.46	0.62	**0.85**
Mts	0.75	0.81	**1.00**
^4^Oth	0.57	0.71	**0.93**
**Precision for:**			
Adc	0.57	0.79	**0.90**
Sqc	0.50	0.67	**0.94**
Mts	0.73	0.79	**1.00**
Oth	0.76	0.91	**0.94**
Kappa	0.50	0.71	**0.89**

*^1^CT-based radiomics (with Random Forest classification). ^2^PET-based radiomics (with Random Forest classification). ^3^The combination of CT- and PET-based radiomics (with Sequential minimal optimization classification). ^4^Other primary lung cancer types. The best performance metrics for each classification are highlighted in bold.*

## Discussion

The CPR models, both binary and four-class classifiers, are reliable to diagnose Pre, Mts, Adc, and Sqc according to the model performance on the test set. In practical application, in order to improve accuracy and reduce run time, we suggest using the four-class CPR model for initial identification and then using the binary models for confirmation. This model can not only help non-invasive diagnosis and support individualized treatment but also can be used as household equipment as long as there are CT and PET images.

Standardized uptake values (SUV) can quantify the differences between repeated measurements, between different scanners, as well as between centers in multicenter trials of PET images (7). It also has good repeatability and reproducibility for radiomics analysis. Kurtosis reflects the shape of the gray-level distribution (peaked or flat) relative to a normal distribution, and Skewness is the asymmetry of the gray-level distribution in the histogram. The four features not only have good repeatability and reproducibility but also have a great classification ability for lung cancer histological subtypes.

Many studies have shown that radiomics features have great potential to be the maker for tumor phenotype (8–17), and found Adc can be differentiated from Sqc by radiomics (17–23). However, The data sets of those studies only included Adc and Sqc, that is to say, the accuracy of those models will be affected by other histological subtypes of lung cancer.

In this study, lung cancer patients with various histological subtypes were included in the patient cohorts. We used stratified random sampling to balance the covariates. In feature selection, we selected 2 CTR features, Skewness and Kurtosis (6) based on histogram, and 2 PETR features, SUVmean and SUVmax (7), with high reproducibility for slice thickness condition changes. The study of stability and reproducibility of the radiomics features (6, 7, 24–31) shows multiple parameter changes (e.g., slice thickness) in general produces greater measurement errors. In this case, the selected 4 features only have good reproducibility against slice thickness. This is also consistent with the studies of Meyer et al. (32) and Sosna (33), who found fewer reproducible radiomic features mean better reproducibility within the same patient. In model selection, both RF and SMO have good robustness and generalization ability.

There are some limitations. First, applying the proposed CPR models should follow the same imaging parameters. Second, CPR models need external validation. Last, the data set we used was from public data sets, so we can not accurately estimate the size and direction of systematic bias.

In conclusion, the proposed CPR models can be used to obtain valid predictions of histological subtypes in lung cancer patients, assisting in diagnosis and shortening the time to diagnostic.

## Data Availability Statement

The raw data supporting the conclusions of this article will be made available by the authors, without undue reservation.

## Author Contributions

MY conceived and designed the study, collected image data, performed the analysis, and wrote the manuscript. WW reviewed the manuscript and acquired the funding. All authors approved the final manuscript.

## Conflict of Interest

The authors declare that the research was conducted in the absence of any commercial or financial relationships that could be construed as a potential conflict of interest.
